# The Past, Present and Future of Porcelain Inlays

**Published:** 1904-07

**Authors:** Charles C. Patten

**Affiliations:** Boston.


### THE PAST, PRESENT AND FUTURE OF PORCELAIN INLAYS.

By Charles C. Patten, D. D. S., Boston.

(Written for *The American Journal of Dental Science.*)

The use of porcelain inlays as a means of checking the ravages of caries in the natural teeth is just now passing through a critical stage. We have arrived at this period after several years of enthusiasm and widespread interest. For at least four or five years back no dental convention or society meeting could call its program complete without one or more clinics or papers for the demonstration of porcelain in some form or other. The main point of interest has been concentrated upon its application as a cavity stopper.

There are still a very great many doubting Thomases pointing the finger of scorn and predicting for it a future of innocuous desuetude. New ideas as well as methods generally meet with considerable opposition from one source or another but it usually comes from people who have condemned it theoretically, not having given it a practical trial or having given it a trial failed for want of skill.

We would not think of condemning the church or even Christian religion just because some church members who profess to be Christians prove to be black sheep.

No more should we condemn porcelain because failures result from carelessness or inexperience. All kinds of operative dentistry may be subjected to abuse of some form, but it does not prove the entire absence of good when rightly used. Bridge and crown work, for instance, have been most abominally abused and yet no one can deny their advantages when used with judgment. Recklessness has no doubt been shown in the use of porcelain, and while it does to a certain extent injure its standing it will in no way influence the competent operator, unless it be to stimulate him to climb to a still higher level in his operative skill. The incompetent or unskillful ones will work out their own execution in this line of work. Porcelain will find its rightful standing within the next few years.

We have looked upon all kinds of operators, all kinds of work, all kinds of methods and materials and listened to all kinds of ideas and suggestions as to which method and material was superior and the reason of its superiority, but every individual must be his own supreme judge as to what method he will pursue and whether his results will come up to the standard. Nothing but the very best will pass muster. Poor fillings of other material will appear to be satisfactory for a time but not so with porcelain. Their imperfections are plainly evident at the very beginning. It does not need the hand of time to develop them.

No one would care to predict that the future does not hold concealed within its mighty grip something more nearly perfect than porcelain combined with cement, but it would seem as though the limit had been reached. The far cry now is for some perfectly insoluble cement for securing the porcelain in place in the cavity. At present there is certainly much to be desired in this respect. Perhaps when some great mind successfully solves this question, vital and deeply interesting as it is, the cement alone will meet all requirements and the porcelain can be dispensed with. It is certainly a fact that either the cement or porcelain can most nearly meet the demands for an ideal filling. The porcelain without the medium of cement is of course useless, therefore to cement must be accorded first place.

The ideal qualities in porcelain consist mainly in its possibility for restoring to perfect harmony the appearance of the teeth. This it can do in a manner superior to any other thing now in use. It can also claim non-conductivity as one of its virtues. Otherwise it has little to commend it as a cavity stopper.

Perfect results require a higher degree of skill than can usually be attained by the average operator. It can hardly claim ease of adaptability, one of the requirements of an ideal filling, and owing to its friable nature it is very susceptible to breakage. In a way its field of usefulness is limited. The skill necessary and time consumed preclude its use among a class of patients who are not financially able to sufficiently remunerate a dentist that he may give the very best that is in him. The enthusiasm of youth when we are prodigal of our time and lavish with our efforts that reputation may result is soon succeeded by the hum-drum of daily existence. Under these circumstances one is justified in expecting compensation that will well repay the high degree of skill and nerve force required in this kind of work.

This argument may savor of commercialism to some, but facts are facts.

The universal inclination among porcelain workers of experience leans towards conservatism. The greatest field that opens up for the work is mainly cavities that come within the range of vision. This involves the superior and inferior front teeth and includes the bicuspids and sometimes first molars. Exceptions can be made and include the other molars, but not often. In the mind of the writer the main function will be to restore a harmonious appearance. Unsightly, glaring gold fillings can be replaced by porcelain with most beautiful results.

It possesses other desirable features well worthy consideration. One that permits of especial mention applies to the treatment of buccal or labial cavities extending to or beyond gum margins where filling with gold means no end of discomfort to patient and the results to operator are seldom entirely satisfactory. This class of cases when skilfully filled, while not exactly a "joy forever are things of beauty." The gingival margins take more kindly to porcelain than gold in such places. Perfect cleanliness is more easily secured and maintained. Approximal cavities not involving the occusal surface or incisal edge are equally promising of success. The various other classes with their different conditions are many and cannot be considered in a brief article.

To sum up: porcelain is by no means a fad, but must be accepted by the profession as one of our standard articles. It must be used with consummate judgment and skill and when so used will amply repay both patient and operator in not only its lasting qualities but beautiful appearance.

